# Fractional laser-assisted topical delivery of bleomycin quantified by LC-MS and visualized by MALDI mass spectrometry imaging

**DOI:** 10.1080/10717544.2019.1574937

**Published:** 2019-03-12

**Authors:** Kristoffer K. Hendel, Charlotte Bagger, Uffe H. Olesen, Christian Janfelt, Steen H. Hansen, Merete Haedersdal, Catharina M. Lerche

**Affiliations:** a Department of Dermatology, Bispebjerg University Hospital, Copenhagen, Denmark;; b Department of Pharmacy, University of Copenhagen, Copenhagen, Denmark

**Keywords:** Bleomycin, drug delivery, fractional laser, MALDI-MSI, skin cancer

## Abstract

Bleomycin exhibits antiproliferative effects desirable for use in dermato-oncology but topical use is limited by its 1415 Da molar mass. Ablative fractional laser (AFL)-assisted drug delivery has been shown to enhance drug uptake in skin. The aim of this study was with AFL to deliver bleomycin into skin, quantify uptake, and visualize biodistribution with mass spectrometry. In a Franz diffusion cell study, pig skin samples (*n* = 66) were treated with AFL (*λ* = 10,600 nm), 5% density, and 0, 5, 20, or 80 mJ/microbeam (mb) pulse energies before exposure to bleomycin for 0.5, 4, or 24 h. Bleomycin was quantified in biopsy cryosections at depths of 100, 500, and 1500 µm using high-performance liquid chromatography-mass spectrometry (LC-MS), and drug biodistribution was visualized for 80 mJ/mb samples by matrix assisted laser desorption/ionization mass spectrometry imaging (MALDI-MSI). The pulse energies 5, 20, and 80 mJ/mb resulted in microscopic ablation zones (MAZs) reaching superficial, mid, and deep dermis respectively. Bleomycin was successfully delivered into the skin and deeper MAZs and longer exposure time resulted in higher skin concentrations. After 24 h, AFL exposure resulted in significant amounts of bleomycin throughout all skin layers (≥510 µg/cm^3^, *p* ≤ .002). In comparison, concentrations in intact skin exposed to bleomycin remained below limit of quantification. MALDI-MSI supported the quantitative LC-MS results by visualizing bleomycin biodistribution and revealing high uptake around MAZs with delivery into surrounding skin tissue. In conclusion, topical drug delivery of the large and hydrophilic molecule bleomycin is feasible, promising, and should be explored in an *in vivo* setting.

AFL: Ablative fractional laser;
CZ: Coagulation zone;
LC-MS: High-performance liquid chromatography-mass spectrometry;
LOQ: Limit of quantification;
MALDI-MSI: Matrix assisted laser desorption/ionization mass spectrometry imaging;
MAZ: Microscopic ablation zone;
MAZ-Deep: Microscopic ablation zone reaching deep dermis;
MAZ-Mid: Microscopic ablation zone reaching mid dermis;
MAZ-Supf: Microscopic ablation zone reaching superficial dermis

## Introduction

Pretreatment with ablative fractional lasers (AFL) shows promise to improve topical therapies by enhancing drug delivery through the skin (Haedersdal et al., 2016). AFL targets water and in precise microbeams ablates tissue, resulting in laser channels termed microscopic ablation zones (MAZ), with surrounding vertical coagulation zones (CZ) of variable thickness (Hantash et al., 2007). Drug properties such as high molecular weight (>500 Da) and water solubility pose challenges to uptake through the lipid bilayers in the stratum corneum of intact skin (Morrow et al., 2007; Aulton, 2013). AFL-assisted drug delivery has been shown to facilitate topical drug uptake of anticancer agents including 5-FU (130 Da) (Wenande et al., 2017), cisplatin (300 Da) (Wenande et al., 2018), vismodegib (431 Da) (Olesen et al., 2018), and methotrexate (455 Da) (Taudorf et al., 2015), all of which have substantially smaller masses than bleomycin (1415 Da and 1426 Da for bleomycin A2 and B2, respectively).

Bleomycin is a cytotoxic glycopeptide used in the treatment of a variety of malignant diseases. The drug’s mechanism of action includes intercalation in DNA and chelation with transition metals to induce reactive oxygen species (Saitta et al., 2008; Povirk et al., 1979). With chemical properties including molar masses of 1415–1426 Da and a predicted log*P* of −7.5 (Kim et al., 2016), bleomycin cannot cross the skin barrier unassisted to any significant degree.

Bleomycin is approved for systemic use in the treatment of squamous cell carcinoma, lymphoma, and testicular cancer (Saitta et al., 2008), and has been used off-label with intralesional administration for a wide variety of malignant and nonmalignant conditions in dermatology, e.g. basal-cell carcinoma (Glass et al., 1997) and keloids (Berman et al., 2017). AFL-assisted drug delivery of bleomycin may be an alternative to intravenous and intralesional administration, to enable a homogenous biodistribution in skin, and minimize systemic effects.

We aim to explore topical delivery of the large and hydrophilic drug bleomycin after AFL delivery, quantify skin uptake with high-performance liquid chromatography-mass spectrometry (LC-MS), and visualize drug biodistribution in superficial (100 µm), mid (500 µm), and deep (1500 µm) dermis using matrix assisted laser desorption/ionization mass spectrometry imaging (MALDI-MSI).

## Materials and methods

### Study design

Delivery of bleomycin by AFL treatment, reaching three different laser channel depths, was examined in an *in vitro* Franz diffusion cell model. Pig skin samples were exposed to bleomycin for 0.5, 4, or 24 h. A total of eleven interventions were tested in six repetitions (*n* = 66) including non-AFL bleomycin interventions and saline controls (Table 1). LC-MS was used to quantitate bleomycin in cryosections from superficial, mid, and deep dermis (100, 500, and 1500 µm depths) along with receiver and donor fluids. MALDI-MSI imaging visualized cutaneous bleomycin biodistribution (100, 500, and 1500 µm depths) in skin samples treated with the deepest laser channels.

**Table 1.** Study design overview.

Interventions	*n*	MAZ^a^	Drug exposure time (hours)	Bleomycin	Saline	MALDI imaging^a^	LC-MS^b^ (n)
1	6	MAZ-Supf	0.5	+	–	–	30
2	6	MAZ-Supf	4	+	–	–	30
3	6	MAZ-Supf	24	+	–	–	30
4	6	MAZ-Mid	0.5	+	–	–	30
5	6	MAZ-Mid	4	+	–	–	30
6	6	MAZ-Mid	24	+	–	–	30
7	6	MAZ-Deep	0.5	+	–	+	30
8	6	MAZ-Deep	4	+	–	+	30
9	6	MAZ-Deep	24	+	–	+	30
10	6	MAZ-Deep	24	–	+	+	30
11	6	No MAZ	24	+	–	–	30
Total	66						330

a Matrix assisted laser desorption/ionization (MALDI) imaging was performed on a single sample per intervention.

b Cryosections (100, 500, and 1500 µm) were quantified along with the donor and receiver with high-performance liquid chromatography-mass spectrometry (LC-MS).

MAZ: Microscopic ablation zone.

MAZ-Supf: Microscopic ablation zone reaching superficial dermis.

MAZ-Mid: Microscopic ablation zone reaching mid dermis.

MAZ-Deep: Microscopic ablation zone reaching deep dermis.

### Skin preparation

Full-thickness skin samples from the flanks of two pigs (female, 46 and 44 kg) were utilized in the study. Skin was excised immediately after euthanasia, trimmed for hair, and relieved of subcutaneous fat. The skin was cut into square pieces of approximately 3 × 3 cm, stored for up to a month at −80 °C and thawed to room temperature at study initiation.

### Ablative fractional laser

AFL treatment was performed with a fractional CO_2_-laser (10,600 nm, DeepFx handpiece, Lumenis Inc., Santa Clara, CA, USA). Laser settings comprised single pulses of 5, 20, or 80 mJ/microbeam (mb) pulse energy at 5% density, generating MAZs reaching superficial-, mid- and deep dermis.

### Franz diffusion cell model

Immediately after AFL treatment, full-thickness skin samples were mounted on a Franz cell model (PermeGear Inc., Hellertown, PA, USA) consisting of a donor and receiver compartment. Skin sample stratum corneum (0.64 cm^2^) faced donor compartments, while receiver compartments contained 5.5–5.8 ml phosphate buffered saline (pH 7.4, 37 °C) and a magnetic stir bar. At baseline, 750 µL of bleomycin sulfate (Baxter, Deerfield, Chicago, USA) (15,000 IU/mL = 7.06 mg/mL) was topically applied to skin samples via donor compartments.

At 0.5, 4, or 24 h diffusion time, skin samples were dismounted, padded dry, and biopsied using an 8-mm punch. A biopsy from each sample was then mounted on Tissue-Tek (Sakura, Alphen, Netherlands) for LC-MS quantitation and laser channel morphology microscopy. The biopsies for MALDI-MSI were mounted on Milli Q water (ELGA CENTRA-R200, ELGA LabWater, Buckinghamshire, United Kingdom). Biopsies were sectioned at skin depths of 100-, 500- and 1500 μm to produce samples of 30 μm thickness for LC-MS and 10 μm for MALDI-MSI. Samples were stored at −80 °C prior to analysis. For laser channel morphology, cryosections were stained with hematoxylin-eosin and photo documented with ProgRes Capture Pro v. 2.8.8 (Jenoptik, Jena, Germany). Laser channel and coagulation-zone dimensions were digitally measured with ImageJ software v. 1.52c (National Institute of Health, Maryland, USA).

### LC-MS

Before LC-MS quantitation, donor and receiver fluids were collected directly from the Franz cells. For skin samples, bleomycin was initially extracted from cryosections for 2 h in 1 mL PBS, rotating vials every 30 min. All samples (250 μL) were then precipitated with 500 µL 2% ZnSO_4_·7H_2_O in 25% methanol, centrifuged at 20,000 rcf for 5 min at 4 °C and the supernatant was analyzed by LC-MS. Standards underwent equivalent analysis.

Quantitative analysis of bleomycin in skin sections, donor, and receiver compartments was performed using an Agilent LC system (Agilent Technologies, 1100 series, Santa Clara, CA, USA) with a binary pump (Agilent 1100 G1312A, Santa Clara, CA, USA) with a MS Single Quadrupole detector, multimode interface. The LC was equipped with a binary solvent delivery system and an autosampler. The injection volume was 10 μL. Separation was achieved at 40 °C using a 10-cm Kinetex XB-C18 column (Phenomenex, Vaerl⊘se, Denmark) with 1.7 µm particle size and 2.1 mm internal diameter. Bleomycin was analyzed under electrospray ionization in positive ion mode with detection in selection ion monitoring mode (SIM) at *m/z* 707.9 and *m/z* 713.3. The mobile phases were (A) 22% acetonitrile in milli Q water + 0.05% heptafluorobutyric acid and (B) acetonitrile. The gradient started at 0% B at 4 min, increased to 90% B at 4–6 min, and returned to 0% B from 6–7 min, sustained until 16 min. The flow rate was 0.25 ml/min, the spray voltage 4000 V, and the drying gas flow rate 9 L/min. The limit of quantification (LOQ) was 75 ng/ml.

### Units and calculations

Skin bleomycin concentrations (µg/cm^3^ and IU/cm^3^) were calculated based on cryosection dimensions of a halved biopsy with a surface area of 0.25 cm^2^ and thickness of 0.003 cm. Receiver concentrations (µg/cm^2^) were calculated based on the exposed skin area of the Franz cell setup (0.64 cm^2^). Percentage of applied concentration present in a cryosection was based on the donor chamber concentration of bleomycin (7.06 mg/mL). In the result section, all concentrations mentioned are median values of 6 repetitions and *p*-values represent the difference between the intervention and control (non-AFL bleomycin treated skin for 24 h).

### Bleomycin imaging in skin

Qualitative determination of the distribution of bleomycin was performed on a Thermo QExactive Orbitrap mass spectrometer equipped with an AP-SMALDI-10 ion source (TransMIT, Giessen, Germany). MALDI imaging was performed on 12 samples and 4 control tissue sections. Prior to analysis, 10 µm sections of frozen tissue, both horizontal at 100, 500 and 1500 µm depth and selected vertical sections, were thawed in a vacuum desiccator and spray-coated with a 30 mg/mL solution of 2,5-dihydroxybenzoic acid in 50:50 methanol/water with 1% trifluoroacetic acid (Hsieh et al., 2007). 300 µL of matrix solution was pneumatically sprayed at a pressure of 2.5 bar, a flow rate of 30 µL/min and a distance between the sample and spray tip of 110 mm to form a homogenous layer of matrix crystals covering an area of approximately 18 mm in diameter. While applying the matrix solution, the sample was rotated at 600 rpm. The mass spectrometer was operated in positive ion mode (scan range *m/z* 400–1600) at mass resolving power of 14,0000@*m/z* 200. A mass accuracy of 1 ppm was ensured by using a matrix peak as lock-mass. The samples were imaged with pixel sizes of 40 µm for horizontal tissue sections and 35 µm for vertical tissue sections. The control tissue sections were analyzed using a pixel size from 100–120 µm, depending on the size of the section. Raw data was converted to imzML (Imaging mass spectrometry markup language) using an imzML converter (Schramm et al., 2012). Images were generated in MSiReader 0.06 using a bin width of 0.02 Da (Robichaud et al., 2013). The image analysis was performed using accurate masses of the two forms of bleomycin in positive ion mode (*m/z* 1415.52609 and 1425.56323) and the tissue was localized on basis of the localization of the endogenous phospholipid PC(34:1) (*m/z* 798.54092).

2,5-dihydroxybenzoic acid and trifluoroacetic acid were purchased from Sigma-Aldrich. Methanol was from ChemSolute/TH Geyer. Milli Q water was produced on a Millipore Direct-Q3 (Merck, Darmstadt, Germany).

### Statistical analyses

Descriptive statistics are presented as medians with interquartile ranges. Testing for statistical significance (5% level), unpaired samples were compared using the non-parametric Kruskal–Wallis and Mann–Whitney *U* tests. If concentrations were below LOQ (75 ng/ml) the LOQ value was used in the calculations. All *p*-values are exact values and two-tailed. Statistical tests were computed in SPSS 24 (IBM Corporation, Armonk, NY, USA). Graphical presentation of data was plotted using GraphPad Prism 7 (GraphPad Software Inc., San Diego, CA, USA).

## Results

### Laser channel dimensions

**Figure 1.**
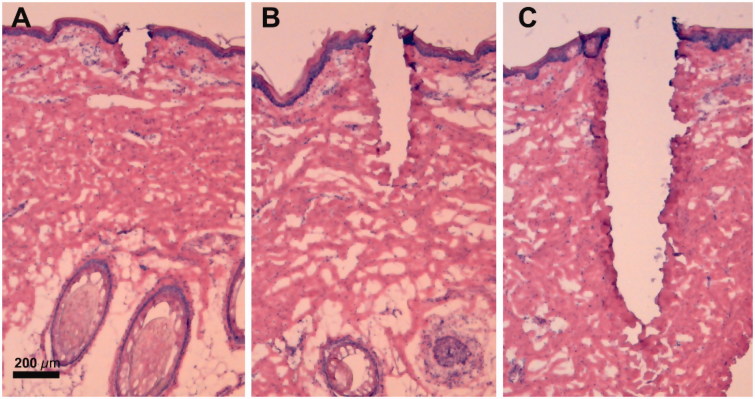
Laser channel morphology and dimensions in HE-stained cryosections. (A) MAZ-Supf, depth: 195 µm (158–219 µm), width: 89 µm (80–101 µm), and coagulation zone: 34 µm (28–35 µm), *n* = 17 channels. (B) MAZ-Mid, depth: 602 µm (513–665 µm), width: 140 µm (118–160 µm), and coagulation zone: 47 µm (43–51 µm), *n* = 21 channels. (C) MAZ-Deep, depth: 1496 µm (1324–1599 µm), width: 251 µm (197–286 µm), and coagulation zone: 65 µm (60–76 µm), *n* = 21 channels. The coagulation-zones bordering the laser channels are visualized by more intense hematoxylin staining than the surrounding tissue. Dimensions are shown as median with interquartile range. Magnification ×40. MAZ-Supf: Microscopic ablation zone reaching superficial dermis; MAZ-Mid: Microscopic ablation zone reaching mid dermis; MAZ-Deep: Microscopic ablation zone reaching deep dermis.

Pulse energies of 5, 20 and 80 mJ/microbeam resulted in median MAZs reaching the superficial- (MAZ-Supf; 195 μm depth), mid- (MAZ-Mid; 602 μm depth) and deep dermis (MAZ-Deep; 1496 μm depth). Histological evaluations of MAZs are shown in Figure 1.

### LC-MS quantified bleomycin concentrations

**Figure 2.**
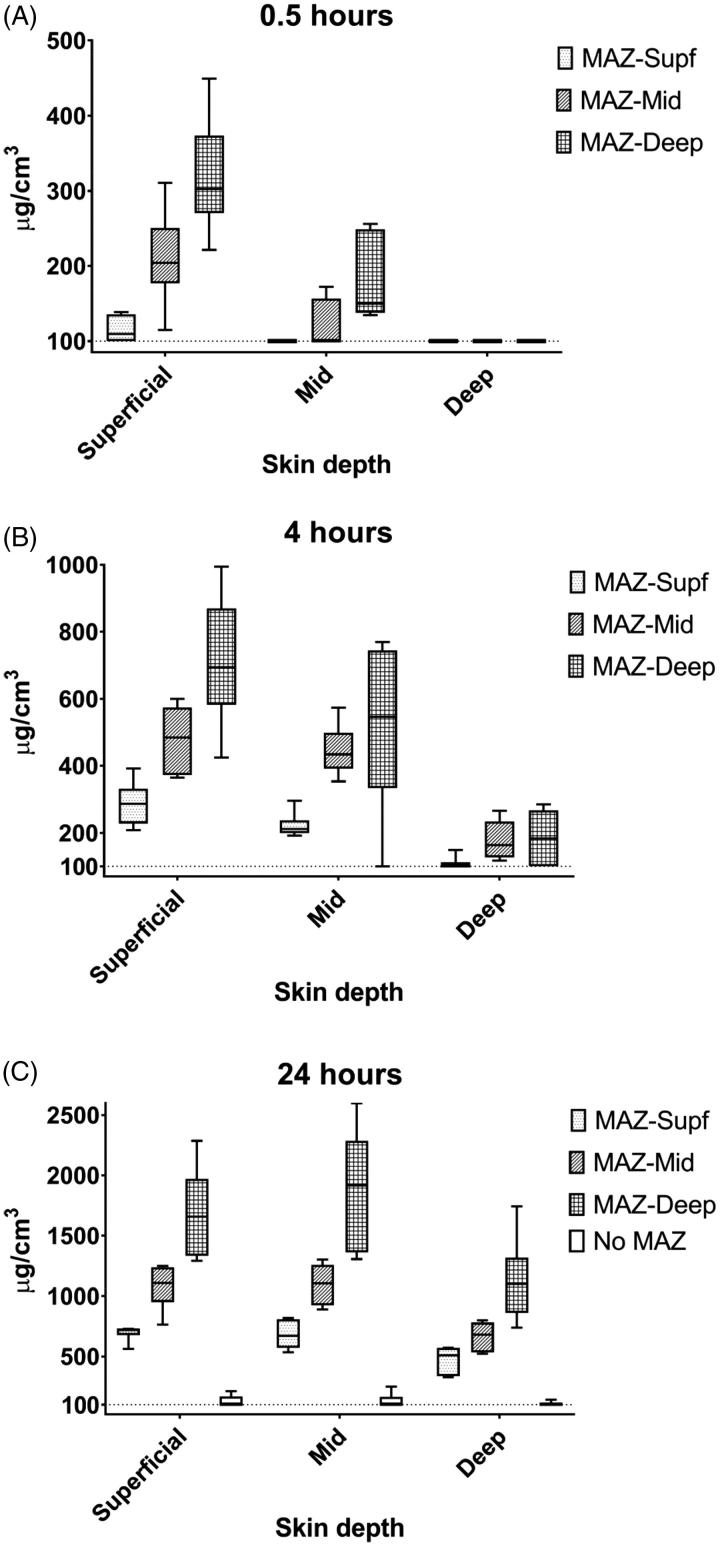
Bleomycin concentrations by exposure time, pulse energies, and skin depth. Boxplots of quantified bleomycin based on median and interquartile range with min/max whiskers for (A) 0.5 h, (B) 4 h, and (C) 24 h drug exposure time. Higher concentrations of bleomycin are seen closer to the skin surface. Higher pulse energies and longer drug exposure times result in higher concentrations of bleomycin. The y-axes are not standardized between diagrams. The dotted lines represent the limit of quantification. MAZ-Supf: Microscopic ablation zone reaching superficial dermis; MAZ-Mid: Microscopic ablation zone reaching mid dermis; MAZ-Deep: Microscopic ablation zone reaching deep dermis.

Overall, drug uptake depended on MAZ depth and exposure time, resulting in higher bleomycin concentrations using deeper MAZs and longer exposure times (Figure 2 and Table 2).

**Table 2.** Quantification and comparison of bleomycin in intervention and control skin.

Intervention	*n*	Sample	Quantified bleomycin concentration (median)				Percentage of applied conc.	Compared with control
			LC-MS^a^	per cm^3^ skin^b^		Receiver^c^		
			ng/mL (IQR)	μg/cm^3^	IU/cm^3^	μg/cm^2^	%	*p*-value
No MAZ								
24 h	6	100 µm	LOQ (LOQ–114.0)	–	–		–	Control
		500 µm	LOQ (LOQ–101.0)	–	–		–	Control
		1500 µm	LOQ (LOQ)	–	–		–	Control
		Receiver	LOQ (LOQ–85.0)			–		
MAZ-Supf								
0.5 h	6	100 µm	82.0 (LOQ–101.0)	109	232		1.6	.818
		500 µm	LOQ (LOQ)	–	–		–	–
		1500 µm	LOQ (LOQ)	–	–		–	–
		Receiver	LOQ (LOQ)			–		
								
4 h	6	100 µm	215.5 (176.0–234.0)	287	610		4.1	.004
		500 µm	158.5 (151.0–164.0)	211	449		3.0	.041
		1500 µm	LOQ (LOQ)	–	–		–	–
		Receiver	LOQ (LOQ)			–		
								
24 h	6	100 µm	541.5 (537.0–545.0)	722	1533		10.2	.002
		500 µm	503.0 (438.0–604.0)	671	1424		9.5	.002
		1500 µm	382.5 (254.0–427.0)	510	1083		7.2	.002
		Receiver	888.5 (714.0–972.0)			7.8		
MAZ-Mid								
0.5 h	6	100 µm	153.0 (148.0–173.0)	204	433		3.0	.026
		500 µm	LOQ (LOQ –114.0)	–	–		–	–
		1500 µm	LOQ (LOQ)	–	–		–	–
		Receiver	LOQ (LOQ)			–		
								
4 h	6	100 µm	363.5 (281.0–424.0)	489	1029		6.9	.002
		500 µm	325.5 (303.0–355.0)	434	922		6.1	.002
		1500 µm	122.50 (97.0–167.0)	163	347		2.3	.015
		Receiver	LOQ (LOQ)			–		
								
24 h	6	100 µm	830.5 (759.0–924.0)	1107	2352		15.7	.002
		500 µm	828.0 (702.0–933.0)	1104	2345		15.6	.002
		1500 µm	510.5 (402.0–582.0)	681	1446		9.6	.002
		Receiver	1317.5 (441.0–2001.0)			11.5		
MAZ-Deep								
0.5 h	6	100 µm	227.0 (215.0–261.0)	303	643		4.3	.002
		500 µm	112.5 (104.0–185.0)	150	319		2.1	.041
		1500 µm	LOQ (LOQ)	–	–		–	–
		Receiver	LOQ (LOQ)			–		
								
4 h	6	100 µm	521.0 (476.0–621.0)	695	1427		9.8	.002
		500 µm	410.0 (309.0–552.0)	547	1161		7.7	.026
		1500 µm	137.5 (LOQ–196.0)	183	389		2.6	.093
		Receiver	LOQ (LOQ)			–		
								
24 h	6	100 µm	1244.0 (1010.0–1400.0)	1659	3522		23.5	.002
		500 µm	1438.5 (1035.0–1635.0)	1918	4073		27.2	.002
		1500 µm	825.0 (676.0–883.0)	1100	2336		15.6	.002
		Receiver	4380.5 (3775.0–5178.0)			38.3		

MAZ: Microscopic ablation zone.

MAZ-Supf: Microscopic ablation zone reaching superficial dermis.

MAZ-Mid: Microscopic ablation zone reaching mid dermis.

MAZ-Deep: Microscopic ablation zone reaching deep.

LOQ: Below limit of quantification (75 ng/mL).

IQR: Interquartile range (Q1-Q3).

a Concentration as measured with LC-MS.

b Cryosection volume is 0.00075 cm^3^ (surface area 0.25 cm^2^ × thickness 0.003 cm).

c Surface area is 0.64 cm^2^.

After 24 h of drug exposure, all three MAZs depths provided significantly increased bleomycin concentrations in all skin layers (*p* ≤ .002). Thus, MAZ-Supf, MAZ-Mid and MAZ-Deep delivered concentrations of 722, 1107, and 1658 µg/cm^3^ bleomycin in superficial dermis, 670, 1104, and 1918 µg/cm^3^ in mid dermis, and 510, 680, and 1100 µg/cm^3^ bleomycin in deep dermis, respectively (Table 2 and Figure 2(C)). In non-AFL-exposed skin, bleomycin concentrations remained below LOQ in all skin layers.

At shorter exposure times, MAZs similarly facilitated significant concentrations of bleomycin in the superficial and mid dermis, though not all MAZ depths resulting in bleomycin in the deep dermis. After 0.5 h of drug exposure, bleomycin reached the superficial dermis with MAZ-Supf (109 µg/cm^3^, *p*=n.s.), MAZ-Mid (204 µg/cm^3^, *p* = .026) and MAZ-Deep (302 µg/cm^3^, *p* = .002). As seen in Table 2 and Figure 2(A), after short-term 0.5 h of drug exposure, only MAZ-Deep delivered bleomycin into mid dermis (150 µg/cm^3^, *p* = .041), and none of the MAZ depths facilitated delivery into deep dermis

After 4 h of topical drug exposure, further increases in bleomycin were observed in the superficial dermis with MAZ-Supf (287 µg/cm^3^, *p* = .004), MAZ-Mid (484 µg/cm^3^, *p* = .002) and MAZ-Deep (695 µg/cm^3^, *p* = .002). Bleomycin was also detected in the mid dermis with MAZ-Supf (211 µg/cm^3^, *p* = .041), MAZ-Mid (434 µg/cm^3^, *p* = .002) and MAZ-Deep (547 µg/cm^3^, *p* = .026). In deep dermis, MAZ-Mid and MAZ-Deep delivered 163 µg/cm^3^ (*p* = .015) and 183 µg/cm^3^ (*p* = .093) bleomycin, respectively (Table 2 and Figure 2(B)), while MAZ-Supf delivery was below LOQ (data not shown).

Bleomycin concentrations in the transdermal receiver chambers were below LOQ or negligible after 0.5 and 4 h of drug exposure with all MAZs. After 24 h, bleomycin reached quantifiable concentrations (Table 2). For all interventions, donor compartments contained more than 75% of the originally applied bleomycin concentration at examined time points (data not shown). Normal saline controls were negative for bleomycin by LC-MS analysis.

### Enhancement ratios

**Figure 3.**
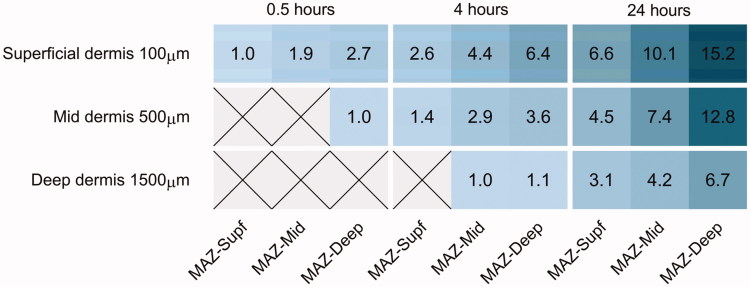
Heatmap of enhancement ratios. Bleomycin concentration enhancement ratios achieved by increasing MAZ depth energy and/or extending the drug exposure time for the superficial, mid, and deep dermis, respectively. For each skin depth separately, the smallest MAZ and drug exposure time setting resulting in a median concentration above the limit of quantification (LOQ) is normalized to 1.00 as a base for enhancement ratios. Thus, values are normalized to Superficial dermis: 82 µg/cm^3^ (MAZ-Supf and 0.5 h), Mid dermis: 112.5 µg/cm^3^ (MAZ-Deep and 0.5 h), Deep dermis: 122.5 µg/cm^3^ (MAZ-Mid and 4 h). A darker hue of blue signifies a higher enhancement ratio. Crossed out cells signify values below LOQ. MAZ-Supf: Microscopic ablation zone reaching superficial dermis; MAZ-Mid: Microscopic ablation zone reaching mid dermis; MAZ-Deep: Microscopic ablation zone reaching deep dermis.

In Figure 3, enhancement ratios depending on MAZ depth and exposure time are shown as a heatmap of investigated skin layers. As illustrated, skin pretreatment with AFL enabled topical delivery of bleomycin with deeper MAZs resulting in enhanced delivery. Longer drug exposure times further enhanced concentrations. Four hours drug exposure time using MAZ-Deep thus offered the same enhancement ratio in superficial skin as 24 h exposure with MAZ-Supf.

### MALDI mass spectrometry imaging

**Figure 4.**
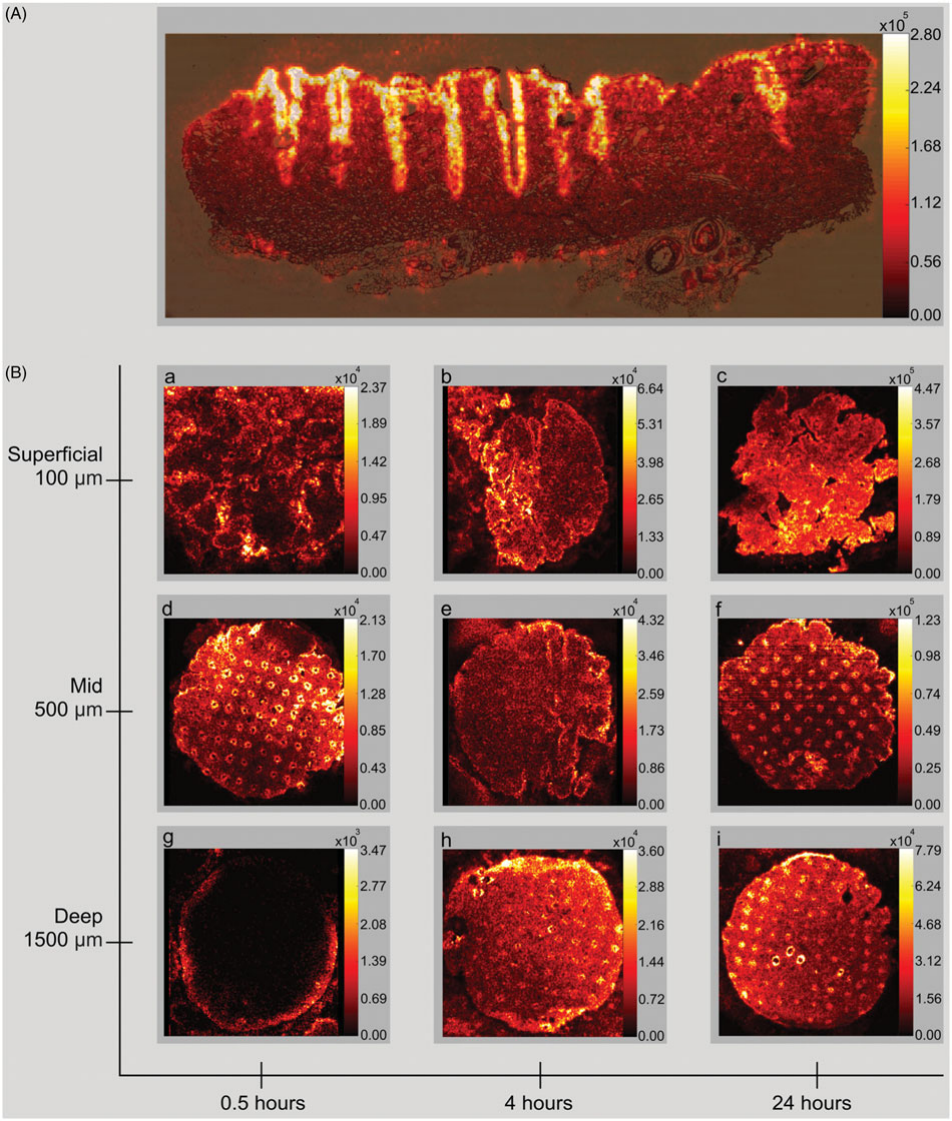
Mass spectrometry imaging. MALDI-MSI of bleomycin B2 (*m/z* 1425.56323) with MAZ-Deep laser channels. (A) Vertically cut skin cryosection after 24 h of topical drug exposure. Laser channels are easily seen with high concentrations of bleomycin (yellow) in the coagulation zones and drug dissemination into the surrounding tissue. (B) Horizontally cut skin cryosections. A trend towards higher concentrations is seen along the x-axis of time, and lower down the y-axis of skin depth. The depicted intensity values for each image are based on maximum bleomycin detection within the individual skin sample and thus cannot be compared inter-individually. MALDI-MSI: Matrix assisted laser desorption/ionization mass spectrometry imaging; MAZ-Deep: Microscopic ablation zone reaching deep dermis.

Qualitative imaging revealed bleomycin concentrating primarily in CZs, though also present in surrounding tissue (Figure 4(A)). Supporting quantitative LC-MS data, a trend of higher concentrations with increased drug exposure time was seen. The highest concentrations of bleomycin were noted in the superficial dermis, with decreasing intensity down through the skin layers.

Drug imaging of the superficial dermis (Figure 4(Ba–Bc)) indicated increasing concentrations of bleomycin over time. Despite poorly defined cryosection outlines, imaging also revealed visible laser channels in analyzed samples.

Mid dermis imaging (Figure 4(Bd–Bf)) showed well-defined skin sample outlines with an organized grid of MAZs. Peak bleomycin concentrations were detected corresponding to CZs and a trend for higher concentrations is seen with increasing drug exposure time.

Deep dermis imaging (Figure 4(Bg–Bi)) showed no visible bleomycin in the center of the 0.5-hour image but the drug is present peripherally (Figure 4(Bg)). This corresponds to the quantitative analysis by LC-MS being below LOQ. The 4 and 24 h images (Figure 4(Bh,Bi)) show well-defined outlines and grid-organized laser channels with a trend of higher bleomycin concentrations with increasing drug exposure time.

No bleomycin was detected in negative control samples with normal saline (data not shown).

## Discussion

The present study is the first assessment of AFL-assisted topical delivery of bleomycin quantified with LC-MS and visualized with MALDI-MSI. Bleomycin was successfully delivered into the deep dermis by AFL while drug detection in non-AFL exposed skin was negligible even at superficial depths (Table 2 and Figure 2(C)). Bleomycin concentrations depended on MAZ depth, and higher concentrations of drug in tissue were achieved using deeper MAZs and longer drug exposure times. As expected, a non-linear relationship between drug uptake and MAZ depth as well as exposure time was observed, indicating a saturation level of bleomycin in the skin similar to data published for other drugs previously delivered by AFL *in vitro*, e.g. 5-FU (Wenande et al., 2017) and methotrexate (Taudorf et al., 2015).

The quantitative LC-MS results are supported by the qualitative MALDI-MSI. Furthermore, the qualitative imaging data provided insight in biodistribution and confirms high uptake of bleomycin in CZs as well as substantial delivery in the surrounding skin tissue. It has been hypothesized that AFL-induced CZs function as a barrier against drug uptake (Erlendsson et al., 2016). However, in the present study, we utilized MALDI imaging to demonstrate substantial biodistribution beyond CZs, similar to what has previously been shown with smaller drugs than bleomycin, e.g. 5-FU (Wenande et al., 2017). Thus, our data is in line with previous research which shows high uptake in CZs with other topically-applied compounds (Haak et al., 2017).

AFL-assisted delivery of bleomycin appears to be a promising candidate for a new topical treatment with uniform and reproducible biodistribution. The AFL modality is associated with less interphysician variance than might be expected with current local treatment methods, e.g. injection delivery. AFL-assisted drug delivery may also enable precision delivery to specific skin depths and might also limit transdermal access to the vascular system and systemic effects; an advantage of local therapy. Although cutaneous biodistribution is highly dependent on drug concentrations and characteristics, a recent *in vivo* study investigating AFL-assisted cisplatin (300 Da) and 5-FU (130 Da) delivery did not find any quantifiable transdermal uptake (Wenande et al., 2018), which indicates that systemic access might be limited depending on the drug and treated area.

AFL-assisted drug delivery in a clinical setting can reduce the need for long drug exposure times. After just 30 min, deep laser channels yielded almost the same drug uptake enhancement ratio in superficial skin as superficial channels after 4 h drug exposure. Thus, increasing MAZ-depth may dramatically reduce drug exposure time.

Compared with reported IC50 test results, our data suggests that AFL-assisted drug delivery of bleomycin can provide clinically relevant concentrations with all tested MAZs. Two studies listed in the Genomics in Drug Sensitivity Cancer (GDSC) project database differ greatly in their IC50 test results for bleomycin on a wide range of cell lines. For the often-used SCC-25 cell line, reported values were 0.97 µg/cm^3^ (0.69 µM) and 219 µg/cm^3^ (155 µM), respectively (Yang et al., 2012).

Another study on the same cell line suggests 15.0 µg/cm^3^ (10.7 µM) after 24 h (Olesen et al., 2017a). Importantly, all three values can easily be reached using AFL under *in vitro* conditions.

We used the standard vehicle for bleomycin for our Franz diffusion cell study, an aqueous saline solution. Topical application in the clinic may benefit from a more viscous formulation in order to adhere securely on the skin for the duration of the treatment, e.g. a gel-based vehicle. In a recent study, we compared different types of vehicles for use with laser-assisted delivery and found that a gel-based vehicle enters the skin almost as effectively as a liquid solution (Olesen et al., 2017b). Future *in vivo* studies might investigate bleomycin’s efficacy and compatibility with different vehicles compared with normal saline. Other factors including drug loading in skin (Karadzovska et al., 2012), closing of laser channels over time (Banzhaf et al., 2017), and vascular wash-out effects also represent areas of further investigation, though best studied *in vivo* (Oni et al., 2012; Ibrahim et al., 2018).

A limitation of this *in vitro* study represents the almost infinite supply of bleomycin dissolved in normal saline available to the skin during exposure. This does not necessarily translate to clinical settings, but may emulate continuous application under occlusion during a similar time-frame in the clinic. Furthermore, Figures 4(Bg–Bi) suggest that punch biopsy can cause depression of peripheral tissue from the skin surface downwards, resulting in cryosections from deep skin compartments displaying tissue from more superficial layers in their outer rims. Higher drug concentrations would in consequence be visualized in the periphery of the cryosections, a phenomenon similarly observable in earlier studies (Wenande et al., 2017). Finally, a few samples of non-AFL-exposed skin were shown to contain bleomycin. However, this finding most likely reflects inadvertent contamination during handling and these low concentrations near the LOQ had a limited impact on our findings.

In conclusion, we show for the first time that AFL-assisted topical drug delivery of the large hydrophilic molecule bleomycin is feasible. Cutaneous bleomycin concentrations can be increased by using deeper MAZs and longer exposure times. Imaging further reflected the quantitative LC-MS data by visualizing bleomycin distributed in CZs and surrounding tissue, as well as higher drug concentrations associated with increased exposure time. AFL-assisted bleomycin delivery appears to be a promising candidate for future *in vivo* studies.

## References

[CIT0001] Aulton ME (2013). Aulton's pharmaceutics: the design and manufacture of medicines. 4th ed Edinburgh: Churchill Livingstone.

[CIT0002] Banzhaf CA, Thaysen-Petersen D, Bay C, et al. (2017). Fractional laser-assisted drug uptake: impact of time-related topical application to achieve enhanced delivery. Lasers Surg Med 49:348–54. 27885682 10.1002/lsm.22610

[CIT0003] Berman B, Maderal A, Raphael B (2017). Keloids and hypertrophic scars: pathophysiology, classification, and treatment. Dermatol Surg 43:S3–s18. 27347634 10.1097/DSS.0000000000000819

[CIT0004] Erlendsson AMKE, Doukas AG, Wang Y, et al. (2016). Merete thermal damage impedes fractional laser-assisted drug delivery [abstract 62]. Lasers Surg Med 48:22.

[CIT0005] Glass LF, Jaroszeski M, Gilbert R, et al. (1997). Intralesional bleomycin-mediated electrochemotherapy in 20 patients with basal cell carcinoma. J Am Acad Dermatol 37:596–9. 9344200 10.1016/s0190-9622(97)70178-6

[CIT0006] Haak CS, Hannibal J, Paasch U, et al. (2017). Laser-induced thermal coagulation enhances skin uptake of topically applied compounds. Lasers Surg Med 49:582–91. 28181673 10.1002/lsm.22642

[CIT0007] Haedersdal M, Erlendsson AM, Paasch U, Anderson RR (2016). Translational medicine in the field of ablative fractional laser (AFXL)-assisted drug delivery: a critical review from basics to current clinical status. J Am Acad Dermatol 74:981–1004. 26936299 10.1016/j.jaad.2015.12.008

[CIT0008] Hantash BM, Bedi VP, Chan KF, Zachary CB (2007). Ex vivo histological characterization of a novel ablative fractional resurfacing device. Lasers Surg Med 39:87–95. 17115384 10.1002/lsm.20405

[CIT0009] Hsieh Y, Chen J, Korfmacher WA (2007). Mapping pharmaceuticals in tissues using MALDI imaging mass spectrometry. J Pharmacol Toxicol Methods 55:193–200. 16919485 10.1016/j.vascn.2006.06.004

[CIT0010] Ibrahim O, Wenande E, Hogan S, et al. (2018). Challenges to laser-assisted drug delivery: applying theory to clinical practice. Lasers Surg Med 50:20–7. 29154501 10.1002/lsm.22769

[CIT0011] Karadzovska D, Brooks JD, Riviere JE (2012). Experimental factors affecting in vitro absorption of six model compounds across porcine skin. Toxicol In Vitro 26:1191–8. 22750544 10.1016/j.tiv.2012.06.009

[CIT0012] Kim S, Thiessen PA, Bolton EE, et al. (2016). PubChem substance and compound databases. Nucleic Acids Res 44:D1202–13. 26400175 10.1093/nar/gkv951 PMC4702940

[CIT0013] Morrow DI, Garland MJ, McCarron PA, et al. (2007). Innovative drug delivery strategies for topical photodynamic therapy using porphyrin precursors. J Environ Pathol Toxicol Oncol 26:105–16. 17725536 10.1615/jenvironpatholtoxicoloncol.v26.i2.50

[CIT0014] Olesen UH, Bojesen S, Gehl J, Haedersdal M (2017a). Anticancer drugs and the regulation of Hedgehog genes GLI1 and PTCH1, a comparative study in nonmelanoma skin cancer cell lines. *Anticancer Drugs *28:1106–17. 10.1097/CAD.0000000000000551 28799948

[CIT0015] Olesen UH, Clergeaud G, Lerche CM, et al. (2018). Topical delivery of vismodegib using ablative fractional laser and micro-emulsion formulation in vitro. *Lasers Surg Med* 51:79–87. 10.1002/lsm.23013 30152536

[CIT0016] Olesen UH, Mogensen M, Haedersdal M (2017b). Vehicle type affects filling of fractional laser-ablated channels imaged by optical coherence tomography. Lasers Med Sci 32:679–84. 28213875 10.1007/s10103-017-2168-z

[CIT0017] Oni G, Brown SA, Kenkel JM (2012). Can fractional lasers enhance transdermal absorption of topical lidocaine in an in vivo animal model? Lasers Surg Med 44:168–74. 22302761 10.1002/lsm.21130

[CIT0018] Povirk LF, Hogan M, Dattagupta N (1979). Binding of bleomycin to DNA: intercalation of the bithiazole rings. Biochemistry 18:96–101. 84680 10.1021/bi00568a015

[CIT0019] Robichaud G, Garrard KP, Barry JA, Muddiman DC (2013). MSiReader: an open-source interface to view and analyze high resolving power MS imaging files on MATLAB platform. J Am Soc Mass Spectrom 24:718–21. 23536269 10.1007/s13361-013-0607-z PMC3693088

[CIT0020] Saitta P, Krishnamurthy K, Brown LH (2008). Bleomycin in dermatology: a review of intralesional applications. Dermatol Surg 34:1299–313. 18616538 10.1111/j.1524-4725.2008.34281.x

[CIT0021] Schramm T, Hester A, Klinkert I, et al. (2012). imzML–a common data format for the flexible exchange and processing of mass spectrometry imaging data. J Proteomics 75:5106–10. 22842151 10.1016/j.jprot.2012.07.026

[CIT0022] Taudorf EH, Lerche CM, Vissing AC, et al. (2015). Topically applied methotrexate is rapidly delivered into skin by fractional laser ablation. Expert Opin Drug Deliv 12:1059–69. 25893560 10.1517/17425247.2015.1031216

[CIT0023] Wenande E, Olesen UH, Nielsen MM, et al. (2017). Fractional laser-assisted topical delivery leads to enhanced, accelerated and deeper cutaneous 5-fluorouracil uptake. Expert Opin Drug Deliv 14:307–17. 27835937 10.1080/17425247.2017.1260119

[CIT0024] Wenande E, Tam J, Bhayana B, et al. (2018). Laser-assisted delivery of synergistic combination chemotherapy in in vivo skin. *J Control Release* 275:242–53. 10.1016/j.jconrel.2018.02.019 29454062

[CIT0025] Yang W, Soares J, Greninger P, et al. (2012). Genomics of Drug Sensitivity in Cancer (GDSC): a resource for therapeutic biomarker discovery in cancer cells. Nucleic Acids Res 41:D955–61. 23180760 10.1093/nar/gks1111 PMC3531057

